# Wearable Assessment of Dynamic Trunk Sway Reveals Directional Balance Adaptations During Sling-Assisted Walking After Stroke

**DOI:** 10.3390/jcm15156104

**Published:** 2026-08-05

**Authors:** Begum Yalcin, Yiğit Can Gökhan, Hülya Şirzai, Güneş Yavuzer, Hande Argunsah

**Affiliations:** 1 Department of Biomedical Engineering, Graduate School of Natural and Applied Sciences, Acibadem Mehmet Ali Aydinlar University, Istanbul 34752, Türkiye; begum.yalcin@acibadem.edu.tr; 2 Department of Computer Engineering, Faculty of Engineering and Natural Sciences, Acibadem Mehmet Ali Aydinlar University, Istanbul 34752, Türkiye; 3 Department of Biomedical Engineering, Faculty of Engineering and Natural Sciences, Acibadem Mehmet Ali Aydinlar University, Istanbul 34752, Türkiye; yigit.gokhan@live.acibadem.edu.tr; 4 Romatem Move Physical Therapy and Rehabilitation Hospital, Istanbul 34640, Türkiye; hulyasirzai@romatem.com (H.Ş.); gunesyavuzer@romatem.com (G.Y.)

**Keywords:** trunk sway, dynamic balance, stroke rehabilitation, arm sling, postural control, neurological rehabilitation

## Abstract

**Background:** Quantitative assessment of dynamic balance remains challenging in clinical practice. Wearable inertial measurement unit (IMU)-based technologies offer an objective and accessible approach for monitoring postural control. This study aimed to develop and preliminarily validate a wearable IMU-based trunk sway monitoring system (SwayTracker) and investigate trunk sway characteristics in stroke patients walking with and without arm sling support. **Methods:** A sternum-mounted IMU system was developed to quantify dynamic trunk sway during walking. Fifteen healthy adults and fourteen stroke patients participated. The healthy participants established normative reference values, whereas the stroke patients completed walking trials with and without arm sling support. Trunk sway was quantified using anteroposterior (AP) and mediolateral (ML) deviations and a polar-coordinate-based sway model. **Results:** The healthy reference cohort exhibited a mean trunk sway magnitude (radius) of 8.20 ± 3.16°. The stroke patients demonstrated greater sway during unsupported (15.36 ± 5.83°) and sling-assisted walking (15.35 ± 4.59°). Although overall sway magnitude remained unchanged, the mean sway direction shifted from 69.49° to 110.20°, indicating a redistribution of trunk sway from the anterior-right toward the anterior-left quadrant. Forward sway remained the dominant AP component, whereas ML sway shifted from predominantly rightward to leftward with sling use. **Conclusions:** SwayTracker provides a feasible method for objective assessment of dynamic trunk sway during walking. The stroke patients exhibited increased sway magnitude and altered directional organization compared with healthy individuals. Arm sling use primarily modified ML postural compensation patterns rather than reducing overall trunk sway, highlighting the potential of wearable trunk sway monitoring for gait and balance assessment in neurological rehabilitation.

## 1. Introduction

Balance control during walking is a complex motor function requiring the integration of sensory, neuromuscular, and biomechanical mechanisms to maintain postural control and safe locomotion. Dynamic balance impairments are associated with increased fall risk, reduced mobility, and decreased functional independence, particularly in neurological populations such as stroke patients. Although clinical balance assessments such as the Berg Balance Scale (BBS), Timed Up and Go (TUG), and Romberg-based tests provide valuable functional information, these methods remain limited in terms of quantitative sensitivity and objective longitudinal monitoring of postural control performance [1].

Traditional laboratory-based gait and balance analysis systems, including motion capture systems and force platforms, provide highly accurate biomechanical data but are expensive, stationary, and difficult to integrate into routine clinical practice. Consequently, wearable inertial measurement unit (IMU)-based systems have emerged as promising tools for objective, portable, and continuous balance assessment during functional activities. Recent systematic reviews have highlighted the growing clinical utility of wearable sensor technologies for objective fall-risk assessment, gait analysis, and rehabilitation monitoring, owing to their ability to provide continuous, quantitative, and ecologically valid measurements of human movement [2,3,4].

Previous studies have shown that trunk-mounted accelerometry can reliably characterize postural sway behavior and balance control. Moe-Nilssen and Helbostad (2002) demonstrated that trunk accelerometry provides objective representation of postural control during standing tasks [5], while subsequent reviews reported that wearable IMU systems can reliably capture anteroposterior (AP) and mediolateral (ML) sway dynamics during functional balance activities [6]. Furthermore, systematic evaluations have demonstrated acceptable validity and reliability of IMU-derived sway parameters for static and dynamic balance assessment applications [7]. Comparative studies additionally reported clinically acceptable agreement between IMU-based measurements and reference laboratory systems using intraclass correlation coefficients (ICC), coefficients of variation (CV), and standard error of measurement (SEM) metrics [8]. These findings support the use of wearable IMU systems as clinically scalable tools capable of providing objective and repeatable postural control measurements outside laboratory environments.

Several wearable systems have previously been developed for balance monitoring and gait analysis. The iSway [9] system utilizes a lower trunk accelerometer to quantify postural sway characteristics, whereas Vertiguard-RT [10] was developed as a wearable vibrotactile feedback system for balance rehabilitation. However, these systems mainly focus on static balance analysis or feedback-oriented rehabilitation rather than continuous dynamic trunk sway monitoring during functional overground walking. In addition, neither system was specifically designed to investigate trunk sway adaptations under different functional walking conditions such as upper extremity stabilization strategies in stroke patients. Compared to these previously proposed systems, the present wearable IMU-based platform was specifically designed for continuous dynamic trunk sway assessment during functional gait tasks using a lightweight wireless architecture. The proposed system enables real-time monitoring of trunk orientation changes during overground walking while providing quantitative analysis of mediolateral and anteroposterior sway behavior under clinically relevant walking conditions.

Stroke-related motor impairments frequently result in gait instability, impaired trunk control, postural asymmetry, and increased fall risk. Following stroke, upper extremity dysfunction may substantially impair balance control and walking performance due to the reduced postural and biomechanical contribution of the hemiparetic side. Loss of natural arm swing, shoulder subluxation, and upper extremity flaccidity have been associated with trunk asymmetry, impaired weight transfer, and compensatory movement strategies during gait, ultimately limiting functional mobility and increasing fall risk.

Arm sling systems are commonly prescribed to support the hemiparetic upper extremity and reduce shoulder subluxation after stroke; however, their effects on gait and balance remain inconclusive. Previous studies have primarily evaluated arm sling use using clinical balance assessments and spatiotemporal gait parameters, reporting inconsistent effects on postural control despite occasional improvements in walking performance. Although several investigations have demonstrated changes in walking speed, cadence, stride characteristics, or clinical balance scores following arm sling application [11,12,13,14,15,16,17], these outcome measures provide only indirect information about trunk control during gait. Quantitative analyses of trunk sway and directional postural adaptations during walking remain scarce. Consequently, the biomechanical mechanisms through which arm sling use may influence dynamic postural control are still not well understood, providing the rationale for the present study.

Previous studies have primarily focused on clinical balance scales and basic spatiotemporal gait parameters, while quantitative analyses of trunk kinematics, postural sway behavior, and dynamic balance adaptations during walking remain limited. Therefore, wearable motion analysis technologies may provide a more comprehensive understanding of how arm sling use influences postural control and gait biomechanics after stroke. Recent literature has additionally highlighted the growing importance of software-supported analysis and clinically interpretable reporting structures in wearable IMU systems [18,19,20]. Moreover, repeated wearable measurements may support longitudinal rehabilitation monitoring and recovery tracking outside laboratory environments [21]. Our previous work also demonstrated a very high correlation between a sternum-mounted IMU-based balance monitoring system and a reference wireless motion analysis system [22], further supporting the technical validity of sternum-mounted trunk sway assessment approaches.

The present study aimed to develop and preliminarily validate a novel wearable IMU-based trunk sway monitoring system for dynamic balance assessment during walking. In addition to evaluating the technical performance of the wearable system, the study investigated trunk sway characteristics in stroke patients under sling and non-sling walking conditions to determine how upper extremity stabilization influences postural control and dynamic gait stability. Accordingly, this study was designed as a translational methodological investigation integrating wearable system development, technical validation, and preliminary clinical application within a single framework to demonstrate the feasibility of objective trunk sway assessment during walking.

## 2. Materials and Methods

This study was designed as a translational methodological investigation integrating wearable system development, technical validation, and preliminary clinical application within a single experimental framework. Rather than representing independent objectives, these components constitute consecutive stages required to establish the feasibility and clinical applicability of the proposed SwayTracker platform for dynamic trunk sway assessment. Accordingly, this section follows the same translational workflow, beginning with the system architecture and signal-processing procedures, followed by the polar-coordinate-based sway modeling approach, participant characteristics, experimental protocol, and statistical analysis.

### 2.1. System Design and Technical Architecture

**Figure 1:**
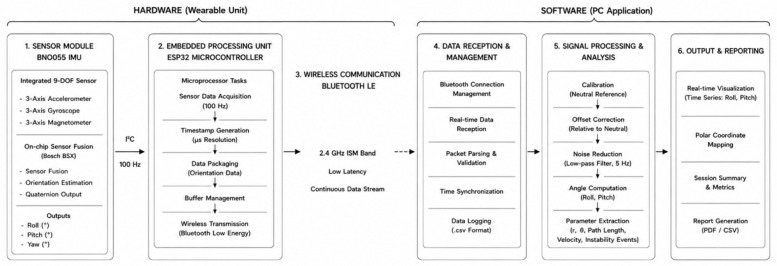
SwayTracker Wearable System Architecture.

SwayTracker is a wearable sensing system developed to quantify dynamic trunk motion and postural sway during activities of daily living (ADL) through continuous monitoring of three-dimensional trunk orientation. Positioned over the sternum, the system integrates inertial sensing, embedded signal processing, wireless communication, real-time visualization, and software-based biomechanical analysis within a unified platform. This architecture enables clinically applicable gait and balance assessment while minimizing setup complexity and preserving natural movement patterns during walking and other daily activities (Figure 1).

The sternum was selected as the sensor placement site because it provides a reliable surrogate of upper-body and whole-body postural behavior during locomotion [23]. Located near the trunk mass center, this anatomical landmark reflects changes in body orientation associated with balance control and weight transfer while reducing soft-tissue artifacts and enabling consistent, reproducible sensor placement across individuals. Consequently, sternum-based measurements offer a practical and clinically meaningful representation of postural regulation during dynamic tasks.

The sensing component is based on the Bosch Sensortec BNO055 (Bosch Sensortec GmbH, Reutlingen, Germany), which incorporates a tri-axial accelerometer, gyroscope, and magnetometer within a compact module. The sensor includes an embedded fusion algorithm that combines acceleration, angular velocity, and magnetic field data to estimate three-dimensional orientation with reduced drift accumulation. Orientation estimates are initially generated in quaternion form and subsequently converted into Euler angles, including roll, pitch, and yaw. Roll represents ML trunk inclination, pitch represents AP trunk inclination, and yaw represents transverse-plane trunk rotation. These orientation parameters are continuously recorded throughout walking tasks and reconstructed as time-series motion trajectories for postural sway analysis.

An ESP32 microcontroller serves as the embedded processing unit responsible for continuous sensor acquisition, timestamp synchronization, packet generation, and wireless communication management. Sensor orientation data were sampled at 100 Hz, providing sufficient temporal resolution for capturing dynamic trunk oscillations and postural adjustments during walking. Data acquired from the BNO055 sensor are transmitted to the microcontroller through an I^2^C interface. The acquired data are subsequently organized into structured packets and transmitted wirelessly to the host computer using Bluetooth communication operating in the 2.4 GHz frequency band.

SwayTracker is powered by a rechargeable 3.7 V Li-ion battery (14,500 form factor; nominal capacity 900 mAh), providing approximately 5 h of continuous operation. The total device weight is approximately 62.08 g. The lightweight wearable structure does not restrict natural trunk or upper-extremity motion during gait assessment.

Real-time orientation data were streamed to a custom-developed desktop application implemented in Python 3.14.6 (Python Software Foundation, Wilmington, DE, USA) specifically for the SwayTracker system. The software environment performs packet integrity verification, signal reconstruction, synchronization, filtering, parameter extraction, real-time visualization, and data storage. The application enables continuous monitoring of roll, pitch, and yaw trajectories together with graphical representation of postural sway behavior. The recorded roll and pitch extrema and the derived radius and theta values were exported for statistical analysis.

For the validation analyses, the recorded roll and pitch extrema together with the derived radius and theta values were exported for statistical analysis. Concurrent validity was evaluated by comparing SwayTracker roll and pitch measurements with simultaneous measurements obtained from the TecnoBody ProKin Trunk Sensor (TecnoBody, Bergamo, Italy) at 11 predefined inclination angles ranging from 0° to 30° in 3° increments. Measurement agreement was assessed using the mean absolute error (MAE), root mean square error (RMSE), mean bias (calculated as SwayTracker minus the reference measurement), and Bland–Altman 95% limits of agreement.

### 2.2. Data Acquisition and Signal Processing

The SwayTracker continuously records trunk orientation data during unrestricted functional activities performed in real-world environments. Owing to its portable wireless architecture, data acquisition can be conducted under dynamic conditions without the need for laboratory-based motion capture systems, dedicated infrastructure, or controlled testing environments. Prior to data acquisition, the sensor undergoes a calibration procedure to establish a neutral reference orientation and minimize baseline offset errors. Calibration is performed while the user maintains a stationary upright standing posture. Raw orientation signals acquired from the IMU sensor are wirelessly transmitted to the custom-developed software environment, where incoming data packets are reconstructed into synchronized time-series datasets. Signal processing procedures include packet integrity verification, removal of initialization artifacts, elimination of transient non-steady-state segments, and segmentation of steady-state walking periods for subsequent biomechanical analysis.

The SwayTracker system quantifies trunk sway behavior using orientation-based angular displacement data derived from the embedded sensor-fusion algorithm. Trunk motion is analyzed in both the sagittal and frontal planes using pitch and roll orientation signals. AP sway is represented by pitch angle (°), whereas ML sway is represented by roll angle (°). For both directions, trunk sway range is calculated as the absolute difference between the maximum and minimum angular displacement values (max–min°) recorded during walking, representing the total excursion amplitude within each movement plane.

Directional deviation is quantified from the absolute magnitudes of forward, backward, rightward, and leftward angular displacements. An absolute-zone output is used to determine the dominant sway direction, whereby the larger absolute deviation within each pair of opposing directions is identified as the predominant postural deviation. This approach facilitated identification of clinically relevant sway tendencies while preventing underestimation of asymmetrical postural behavior caused by cancellation effects between opposing directional excursions.

### 2.3. Polar Coordinate-Based Trunk Sway Modeling

**Figure 2:**
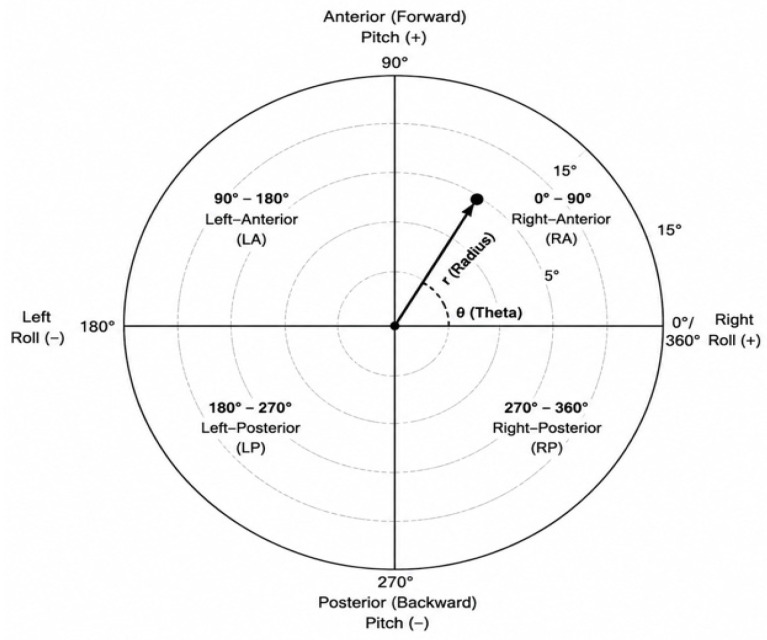
Schematic illustration of the polar coordinate approach used for trunk sway analysis. Roll (ML trunk inclination) and pitch (AP trunk inclination) components obtained from the SwayTracker system are mapped onto a circular coordinate plane to determine both directional orientation and combined sway magnitude. The radius (r) represents the overall sway magnitude, whereas the angular component (θ) defines sway direction within the four directional quadrants.

To quantify overall trunk sway independent of movement direction, ML (roll) and AP (pitch) inclination components obtained from the SwayTracker system are transformed into a polar coordinate framework (Figure 2). Within this model, roll is assigned to the x-axis and pitch to the y-axis, allowing simultaneous characterization of sway magnitude and directional orientation during walking.

The combined sway magnitude is represented by the radius (r) of the polar coordinate system and calculated using the Euclidean distance formula:

$$r=\sqrt{roll^{2}+pitch^{2}}$$

where roll represents ML trunk inclination, pitch represents AP trunk inclination, and r represents the resultant trunk sway magnitude. This parameter reflects the total angular deviation of the trunk from the neutral upright position regardless of direction. Smaller radius values indicate reduced postural sway and greater trunk stability, whereas larger values represent increased multidirectional trunk deviation and reduced postural control.

To determine sway direction, the angular coordinate (θ) was calculated using the four-quadrant inverse tangent function:

$$\theta =atan2\left(pitch,roll\right)$$

Angular values were converted from radians to degrees and normalized to a 0–360° range using:

$$\theta_{deg}=\theta \times \frac{180}{\pi }$$

If $\theta_{deg}<0$, then:

$$\theta_{final}=\theta_{deg}+360,\theta_{deg}<0$$

Within this framework, 0°/360° corresponds to rightward inclination, 90° to anterior inclination, 180° to leftward inclination, and 270° to posterior inclination. The resulting polar-coordinate representation enables simultaneous assessment of sway magnitude and directional tendency, providing a comprehensive description of trunk sway behavior during gait. Combined sway trajectories and directional distributions are subsequently visualized using circular displacement maps generated within the SwayTracker software (Version 1.0) environment. Consequently, each walking trial is represented by two complementary parameters: (i) the combined sway magnitude (radius, *r*), reflecting the overall amount of trunk deviation, and (ii) the directional angle (*θ*), describing the predominant orientation of trunk sway.

### 2.4. Participants

Two participant groups were included in the study: a healthy control group and a stroke group (Table 1). The healthy control group consisted of 15 healthy and relatively young adults (9 females and 6 males) with a mean age of 20.9 ± 1.1 years. All the healthy participants were right-side dominant and had no history of neurological, musculoskeletal, vestibular, or balance-related disorders affecting gait or postural control. The participants were recruited from the university population and completed walking assessments at their self-selected comfortable walking speed. The mean self-selected walking speed of the healthy group was 2.8 ± 0.8 km/h. The healthy cohort was included to establish a methodological reference dataset for trunk sway characterization rather than to serve as an age-matched control group for direct statistical comparison with the stroke patients.

The stroke group (*n* = 14, 4 females and 10 males; mean age = 57.6 ± 9.5 years) consisted of individuals with a history of unilateral stroke presenting with hemiparetic upper extremity involvement, impaired arm swing, and shoulder instability requiring upper extremity support during ambulation. An a priori power analysis was performed using G*Power (Version 3.1.9.7, Heinrich Heine University Düsseldorf, Germany) for the paired comparisons between walking conditions. The analysis was based on a two-tailed paired-samples t-test assuming an α level of 0.05, a desired statistical power (1 − β) of 0.95, and an expected moderate-to-large effect size (Cohen’s d = 0.75). The analysis indicated that a minimum of 14 participants would be required. Accordingly, the final stroke cohort (*n* = 14) satisfied the predefined sample size requirement for the planned within-subject analyses.

The stroke patients were significantly older than healthy controls (*p* < 0.001) and exhibited significantly higher BMI values (*p* = 0.049). No significant between-group differences were observed for sex distribution, height, or body weight (*p* > 0.05). Walking speed was substantially lower in the stroke group compared with healthy controls (*p* < 0.001), reflecting impaired gait performance and mobility following stroke.

The stroke participants were recruited from a specialized inpatient rehabilitation clinic and were evaluated under two experimental conditions: walking without arm sling support (WO Sling) and walking while wearing a standardized arm sling (W Sling) positioned on the affected upper extremity. Inclusion criteria for the stroke group included: age between 40 and 75 years; diagnosis of unilateral ischemic or hemorrhagic stroke; stroke duration between 1 week and 24 months; presence of hemiparetic shoulder subluxation or upper extremity flaccidity; ability to tolerate upright standing for at least 30 min; ability to walk independently or with minimal supervision on treadmill and sufficient cognitive and communication abilities to understand verbal instructions and participate in the experimental procedures. Participants were excluded if they had severe lower extremity spasticity, contracture, or pain significantly limiting ambulation; additional orthopedic, neurological, vestibular, or neuromuscular disorders affecting gait; uncontrolled cardiovascular or systemic conditions limiting exercise tolerance; severe cognitive impairment; severe aphasia preventing communication; or inability to safely complete the walking assessments.

Prior to participation, all the individuals received detailed verbal and written information regarding the study procedures and provided written informed consent. The study protocol was approved by the institutional ethics committee (protocol code: 2024-KAEK-37, decision no:2025/0056) and conducted in accordance with the principles of the Declaration of Helsinki.

### 2.5. Experimental Protocol

**Figure 3:**
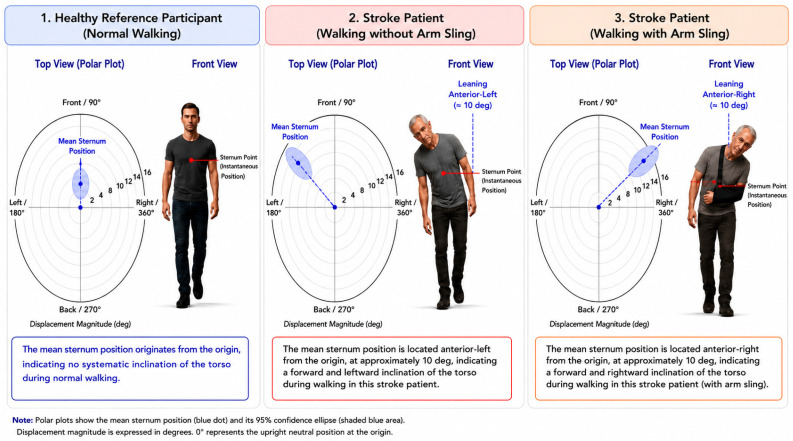
Representative visualization of trunk sway orientation and sternum displacement patterns obtained using the SwayTracker system. The figure demonstrates the integration of polar-coordinate-based displacement mapping with real-time trunk inclination visualization in three representative conditions: (1) Healthy reference participants, (2) WO Sling walking, and (3) W Sling walking. In the top-view polar plots, the blue point represents the mean sternum position, the shaded region indicates the 95% displacement distribution, and the dashed vector illustrates the dominant trunk inclination direction. The front-view images display instantaneous sternum position and body inclination relative to the reference origin. All the directional labels (anterior, posterior, left, and right) refer to the participant’s anatomical directions (patient’s perspective), not the viewer’s perspective. Directional orientation is defined according to the circular coordinate system: anterior (90°), posterior (270°), left (180°), and right (0°/360°).

All the measurements were performed on a treadmill in a controlled indoor environment. The participants were instructed to walk at their self-selected comfortable walking speed to preserve natural gait behavior and minimize externally imposed movement adaptations. Prior to data acquisition, the participants were familiarized with the experimental procedures. Healthy individuals completed a single walking condition without arm sling support to establish normative trunk sway characteristics during typical gait. The stroke patients completed two walking conditions: (1) WO Sling walking and (2) W Sling walking (Figure 3). The sling was individually adjusted by an experienced physiotherapist to ensure reproducible positioning and adequate support of the shoulder and forearm during ambulation. The order of the two walking conditions was randomized to minimize sequence and learning effects.

The stroke participants completed one 10 m walking trial under each experimental condition at a self-selected walking speed. Because the walking task was brief, fatigue was not formally quantified using a standardized fatigue scale. A rest period was provided until the participant reported feeling comfortable to continue, as judged by both the participant and the supervising physiotherapist. Because the walking task consisted of a short 10 m trial, fatigue was not formally assessed using a standardized instrument. All the stroke participants performed the walking trials on the Tecnobody Walker View system (Tecnobody, Bergamo, Italy) at their self-selected comfortable walking speed. A body-weight support harness was applied to ensure participant safety during data acquisition. All the assessments involving the stroke patients were supervised by an experienced physiotherapist, and appropriate safety precautions were implemented throughout the study to minimize fall risk. Rest periods were provided between trials to reduce fatigue-related effects on gait performance and postural control, with additional recovery time allowed when clinically necessary. During all the walking tasks, the participants were instructed to look forward and walk naturally without receiving additional gait-related verbal cues. Throughout all the walking trials, the SwayTracker system continuously recorded trunk orientation and sway-related parameters in real time. The sensor was secured over the sternum using an adjustable elastic strap and remained in the same anatomical position throughout all the experimental conditions to ensure measurement consistency.

Normative reference values for trunk sway behavior were established using data obtained from the healthy control group. Because of the relatively small sample size (*n* = 15), nonparametric 95th percentile (P95) analysis was used to define upper reference limits for the linear sway variables. Reference thresholds were calculated separately for AP sway, ML sway, and the combined sway magnitude (radius, *r*). To account for the potential influence of walking speed on trunk sway behavior, reference values were derived from the healthy participants during both self-selected walking and standardized slow walking (1 km/h). The two walking conditions were analyzed independently, and P95 values were calculated separately for each variable. For each linear parameter, the higher of the two P95 values was adopted as the final upper reference limit to provide a conservative estimate of physiological trunk sway and to avoid underestimating sway variability during slower gait.

Unlike sway magnitude, the directional angle (theta, *θ*) represents circular data and therefore was not characterized using percentile-based thresholds. Instead, healthy directional behavior was summarized using circular descriptive statistics (mean direction and circular standard deviation) together with quadrant-based frequency distributions. These directional characteristics served as normative references for interpreting predominant trunk sway orientation in the stroke patients. The resulting normative reference values were subsequently used to contextualize trunk sway behavior in the stroke patients and to evaluate deviations from healthy balance patterns under WO Sling and W Sling walking conditions. The primary trunk sway outcomes included AP sway range (pitch range), ML sway range (roll range), sway origin deviation, directional maximum angular deviations, dominant sway direction, and combined sway magnitude derived from the polar-coordinate model. Directional deviation analysis was performed to identify asymmetrical trunk motion tendencies and compensatory postural strategies during locomotion. Given the established association between mediolateral instability, impaired dynamic balance, and fall risk in neurological populations, particular emphasis was placed on ML sway behavior during comparisons involving the stroke patients.

### 2.6. Statistical Analysis

Statistical analyses were performed to characterize trunk sway behavior in healthy individuals and to evaluate differences in dynamic postural control between WO Sling and W Sling walking conditions in the stroke patients. Descriptive statistics were calculated for all the outcome variables and were presented as mean ± standard deviation (SD), median, minimum, and maximum values, as appropriate. Because the polar-coordinate analysis generated both a linear magnitude variable (radius, *r*) and a circular directional variable (theta, *θ*), these parameters were analyzed using different statistical approaches. The radius (*r*) was treated as a continuous linear variable and summarized using conventional descriptive statistics. In contrast, the directional angle (*θ*) was analyzed as circular data. Accordingly, group-level directional behavior was summarized using the circular mean direction and circular standard deviation. The circular mean was calculated from the mean sine and cosine components of individual *θ* values, whereas the circular standard deviation was derived from the mean resultant vector length. Directional sway patterns were additionally described using quadrant-based frequency distributions.

The normality of continuous variables was assessed using the Shapiro–Wilk test. Depending on the distribution characteristics, between-group comparisons of participant characteristics were performed using independent-samples *t*-tests or Mann–Whitney *U* tests. Sex distribution was compared using Fisher’s exact test. Within the stroke group, differences between walking with and without arm sling support were evaluated using paired statistical analyses. Paired-samples *t*-tests were applied to normally distributed variables, whereas Wilcoxon signed-rank tests were used for non-normally distributed variables. Comparisons included AP sway, ML sway, directional angle (*θ*), combined sway magnitude (*r*), and other derived trunk sway parameters. A two-sided *p*-value < 0.05 was considered statistically significant. Statistical analyses were performed using IBM SPSS Statistics (version 31.0, IBM Corp., Armonk, NY, USA). Circular statistical analyses were performed using the Circular Statistics Toolbox for MATLAB (CircStat, version 1.21.0.0; Philipp Berens) implemented in MATLAB R2025b (The MathWorks Inc., Natick, MA, USA).

## 3. Results

### 3.1. Concurrent Validation of the SwayTracker System

A preliminary concurrent validation was performed using 11 simultaneous paired measurements acquired at predefined inclination angles (0–30°, 3° increments) in both the roll and pitch planes. For roll, the mean absolute error (MAE) was 1.12°, the root mean square error (RMSE) was 1.15°, and the mean bias (SwayTracker − reference system) was +1.12°, with Bland–Altman 95% limits of agreement ranging from 0.59° to 1.65°. For pitch, the MAE was 1.14°, the RMSE was 1.17°, and the mean bias was +1.14°, with 95% limits of agreement ranging from 0.54° to 1.73°. The maximum absolute difference between SwayTracker and the reference system was 1.60° for both planes. These agreement metrics complement the previously observed strong linear relationships between the two systems (roll: R^2^ = 0.997; pitch: R^2^ = 0.996).

### 3.2. Trunk Sway Characteristics in Healthy Individuals

**Figure 4:**
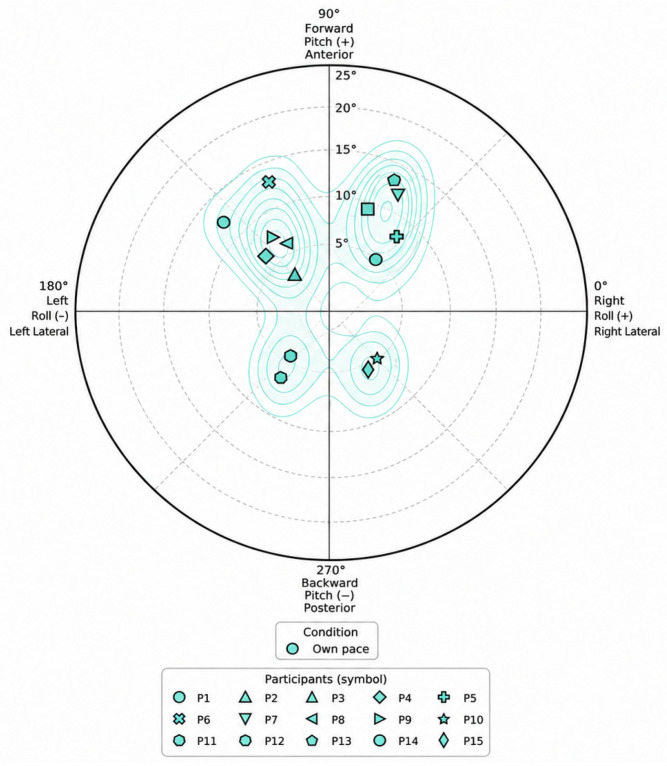
Polar representation of balance profiles in healthy individuals during own-pace walking. Individual healthy participants (P1–P15) are shown as separate symbols according to their composite trunk sway magnitude (radius (r)°) and directional orientation (theta (θ)°) in the polar coordinate plane. The mint-shaded region (“Control Sway Zone”) represents the overall distribution envelope derived from healthy participants, encompassing the range of combined sway magnitudes and directional tendencies observed during normal walking.

The healthy reference dataset provides the normative distribution of trunk sway against which the measurements obtained from the stroke participants can be descriptively contextualized. Polar sway analysis was performed in 15 healthy participants using combined roll and pitch measurements obtained from the SwayTracker system. Individual trunk orientation vectors were transformed into polar coordinates to characterize both sway direction and overall trunk deviation magnitude. The healthy participants demonstrated predominantly anteriorly oriented trunk sway during self-selected walking. Eleven of the fifteen participants were located within the anterior quadrants, whereas four participants exhibited posterior sway orientations. Mean roll and pitch magnitudes were 4.33 ± 1.81° and 7.62 ± 3.17°, respectively, resulting in a mean composite sway radius of 8.93 ± 3.19°, showing that the healthy participants generally remained close to the origin of the polar coordinate map, consistent with limited resultant trunk deviation during normal gait. The circular mean direction of theta was 108.63°, corresponding to an anterior-left resultant sway direction within the polar coordinate system. However, the relatively large circular SD of 72.82° indicated broad directional dispersion among the healthy participants. Therefore, although anterior sway represented the dominant postural tendency during normal walking, healthy individuals also exhibited inter-subject variability in directional sway organization. The polar distribution showed a tendency toward forward-directed trunk motion, indicating that anterior sway represented the dominant postural pattern during normal walking (Figure 4). The healthy participant data were subsequently used to establish normative reference thresholds for trunk sway parameters. These reference values served as the basis for interpreting trunk sway behavior in the stroke patients and for identifying deviations from expected healthy walking patterns. Although healthy individuals exhibited inter-subject variability in sway direction, the majority remained concentrated within a restricted region near the origin of the polar coordinate map.

### 3.3. Trunk Sway Characteristics of Stroke Patients Without Arm Sling Use

Trunk sway behavior was first evaluated during the WO Sling condition to characterize baseline postural control in the stroke patients. Polar-coordinate analysis revealed a predominantly forward-oriented sway pattern, with 10 of 14 participants demonstrating dominant forward pitch deviations and 4 participants demonstrating dominant backward deviations (Table 2). In the ML direction, 9 participants demonstrated a dominant rightward sway, and 5 participants demonstrated a dominant leftward sway. Among the 9 participants with rightward sway, 4 participants presented with right-side-affected hemiparesis, 4 participants with left-side-affected hemiparesis and 1 participant with bilateral hemiparesis. Thus, the rightward ML tendency observed during unsupported walking was not exclusive to patients with right-sided hemiparesis. Consequently, the anterior-right quadrant represented the most frequently observed sway orientation, encompassing 7 participants, followed by the anterior-left (*n* = 3), posterior-left (*n* = 2), and posterior-right (*n* = 2) quadrants.

Mean pitch and roll magnitudes were 12.92 ± 5.98° and 7.77 ± 2.72°, respectively, indicating that trunk sway during WO Sling walking was primarily influenced by anteroposterior motion. At the individual level, pitch magnitude exceeded roll magnitude in 13 of 14 participants, further supporting the predominance of AP sway deviation during WO Sling support. The mean composite sway radius was 15.36 ± 5.83°, nearly twice the value observed in the healthy participants, suggesting substantially greater overall trunk deviation during gait. Considerable inter-individual variability was observed, with radius values ranging from 8.41° to 27.97°. The circular mean direction of theta was 69.49°, corresponding to an anterior-right resultant sway direction within the polar coordinate system. The relatively large circular SD of 79.15° indicated broad directional dispersion across the participants, suggesting that although an anterior-right tendency was evident at the group level, individual trunk sway strategies were heterogeneous during unsupported walking.

**Figure 5:**
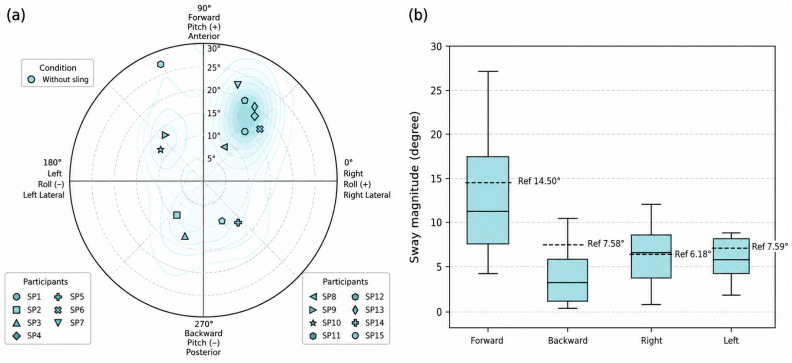
Trunk sway characteristics of stroke patients during WO Sling walking: (**a**) Polar-coordinate representation of individual trunk sway profiles showing composite sway magnitude (radius) and directional orientation (θ) for each participant. The shaded contour indicates the overall distribution of trunk sway across the stroke cohort. (**b**) Distribution of directional sway magnitudes in the forward, backward, rightward, and leftward directions. Dashed horizontal lines represent the corresponding normative reference thresholds (P95) derived from healthy participants. Values exceeding the reference lines indicate trunk sway magnitudes outside the expected healthy range.

The polar-coordinate distribution further illustrates the organization of trunk sway during WO Sling walking (Figure 5a). Most participants were clustered within the anterior-right quadrant, indicating a combined pattern of forward pitch and rightward roll. Although a common anterior-right tendency was evident, the presence of participants across all four quadrants demonstrated substantial heterogeneity in both sway direction and magnitude. The participants located farther from the origin exhibited larger composite trunk deviations, whereas those positioned closer to the center demonstrated better control of trunk motion during gait.

For theta, all 14 paired observations were retained. The circular mean was 74.50° during WO Sling walking (circular SD, 80.33°; mean resultant length, R = 0.374) and 110.20° during W Sling walking (circular SD, 41.97°; R = 0.765). The paired unit-vector analysis did not show a statistically significant group-level directional change (T^2^ = 6.63; F(2,12) = 3.06, *p* = 0.084; exact within-pair permutation *p* = 0.075). An exploratory joint analysis of the paired signed ML and AP components also included all 14 participants. The mean W Sling-minus-WO Sling shift was −5.04° along the ML axis and +2.84° along the AP axis, and the joint two-dimensional change was not statistically significant (T^2^ = 2.89; F(2,12) = 1.33, *p* = 0.300; exact sign-flip *p* = 0.303; Table 3).

Comparison with healthy reference thresholds revealed that several stroke participants exceeded the normative limits for forward and rightward sway (Figure 5b). In contrast, backward and leftward sway generally remained within or close to the healthy reference ranges. These findings indicate that WO Sling walking in the stroke patients was characterized by increased anterior trunk deviation accompanied by a tendency toward right-sided mediolateral compensation. Overall, WO Sling walking was characterized by increased trunk sway magnitude relative to healthy individuals, with a predominant anterior-right directional organization and considerable variability across the stroke patients.

### 3.4. Trunk Sway Characteristics of Stroke Patients with Arm Sling Use

Trunk sway behavior was subsequently evaluated during the W Sling condition to investigate the influence of upper-extremity stabilization on postural control. Polar-coordinate analysis demonstrated that trunk sway remained predominantly forward-oriented, with 13 of 14 participants exhibiting dominant forward pitch deviations and only one participant exhibiting a dominant backward deviation (Table 4). In the mediolateral direction, 10 participants demonstrated dominant leftward sway, whereas 4 participants demonstrated dominant rightward sway. Consequently, the anterior-left quadrant represented the most frequently observed sway orientation during W Sling walking.

Mean pitch and roll magnitudes were 12.34 ± 4.49° and 8.53 ± 3.55°, respectively, indicating that trunk sway continued to be primarily influenced by anteroposterior motion. At the individual level, pitch magnitude exceeded roll magnitude in 10 of 14 participants, further supporting the predominance of AP sway deviation during W Sling support, although ML contribution sway radius was 15.35 ± 4.59°, demonstrating substantial overall trunk deviation during gait. Radius values ranged from 8.42° to 24.38°, reflecting considerable inter-individual variability in trunk control despite sling assistance. The circular mean direction of theta was 110.20°, corresponding to an anterior-left resultant sway direction within the polar coordinate system. The circular SD of 41.97° indicated moderate directional dispersion across the participants, suggesting that although individual variability persisted, trunk sway direction W Sling support was more consistently organized toward the anterior-left quadrant compared with the WO Sling condition.

**Figure 6:**
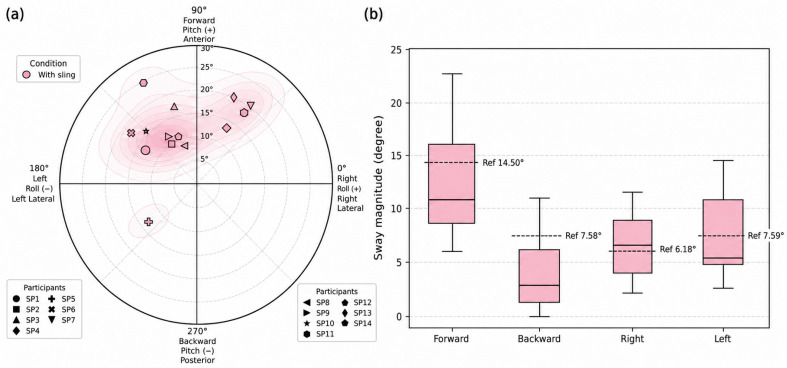
Trunk sway characteristics of stroke patients during W Sling walking: (**a**) Polar-coordinate representation of individual trunk sway profiles showing composite sway magnitude (radius) and directional orientation (θ) for each patient. The shaded contour indicates the overall distribution of trunk sway across the stroke cohort. (**b**) Distribution of directional sway magnitudes in the forward, backward, rightward, and leftward directions. Dashed horizontal lines represent the corresponding normative reference thresholds (P95) derived from healthy participants. Values exceeding the reference thresholds indicate trunk sway magnitudes outside the expected healthy range.

The polar-coordinate distribution further illustrates the organization of trunk sway during W Sling walking (Figure 6a). Most participants clustered within the anterior-left quadrant, indicating a combined pattern of forward pitch and leftward roll. Although a common anterior-left tendency was evident, the spread of participants across different quadrants and radial distances demonstrated substantial variability in both sway direction and magnitude. Comparison with healthy reference thresholds showed that several participants exceeded the normative limits for forward and leftward sway (Figure 6b). In contrast, backward sway generally remained below the healthy reference threshold. These findings suggest that anterior trunk deviation persisted during sling-assisted walking, while mediolateral compensation was more frequently directed toward the left side. Overall, W Sling walking was characterized by a predominantly anterior-left trunk sway pattern. While forward trunk deviation remained the dominant feature, the mediolateral organization of sway shifted toward the left side, suggesting that sling use influenced directional postural compensation strategies rather than substantially reducing overall trunk sway magnitude.

### 3.5. Effect of Arm Sling Use on Trunk Sway Organization

**Figure 7:**
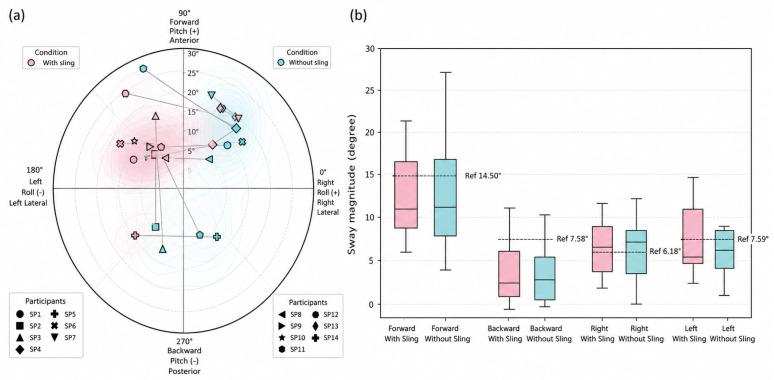
Effect of arm sling use on trunk sway organization in stroke patients: (**a**) Polar-coordinate representation of trunk sway profiles during W Sling (pink) and WO Sling (blue) walking conditions. Individual participants are connected by gray lines to illustrate within-participant changes in sway direction and composite sway magnitude (radius). The shaded contours represent the distribution density of trunk sway under each condition. (**b**) Comparison of directional sway magnitudes between WO and W Sling walking conditions in the forward, backward, rightward, and leftward directions. Dashed horizontal lines indicate the corresponding normative upper reference thresholds (95th percentile, P95) derived from healthy participants.

To evaluate the influence of arm sling support on postural control during walking, trunk sway characteristics were compared between WO Sling and W Sling walking conditions in the stroke patients. The polar-coordinate comparison provided additional insight into the directional organization of trunk sway (Figure 7a). During WO Sling walking, most participants were clustered within the anterior-right quadrant, reflecting a combination of forward pitch and rightward roll. Following arm sling use, the distribution shifted toward the anterior-left quadrant, indicating a combined pattern of forward pitch and leftward roll. This directional shift showed that sling use primarily affected the orientation of trunk sway rather than the magnitude of sway itself. Consistent with this observation, the radial distances of the sway vectors were broadly similar between conditions, indicating comparable composite sway magnitudes. Thus, although arm sling support altered the directional organization of trunk sway, it did not appear to substantially reduce overall trunk deviation at the group level.

The comparison of directional sway magnitudes demonstrated that forward sway remained the dominant trunk sway component in both conditions (Figure 7b). Median forward sway values were comparable between WO Sling and W Sling walking, although several participants exceeded the healthy reference threshold in both conditions. Backward sway remained substantially lower than forward sway and generally below the healthy reference limit, indicating that posterior trunk deviation was not a predominant characteristic of gait in this cohort.

Comparisons between walking with and without sling support were performed using paired-samples analyses (Table 5). The paired differences satisfied the assumption of normality (Shapiro–Wilk, all *p* > 0.05); therefore, paired-samples *t*-tests were applied. No significant differences were observed in mediolateral sway (roll magnitude: 7.77 ± 2.72° vs. 8.53 ± 3.55°, *t*(13) = 0.968, *p* = 0.351), anteroposterior sway (pitch magnitude: 12.92 ± 5.98° vs. 12.34 ± 4.49°, *t*(13) = −0.503, *p* = 0.623), sway direction (theta, *t*(13) = −0.947, *p* = 0.361), or combined sway magnitude (radius: 15.36 ± 5.83° vs. 15.35 ± 4.59°, *t*(13) = −0.005, *p* = 0.996). These findings indicate that arm sling use did not significantly alter the magnitude of trunk sway, although individual changes in sway direction were observed.

The patient-level quadrant transition analysis (Table 6) demonstrated that the effect of arm sling use was primarily reflected in changes in the directional organization of trunk sway rather than in a uniform reduction in sway magnitude. WO sling, the anterior-right quadrant was the most frequently observed orientation, occurring in 7 of 14 patients (50.0%). W Sling walking, the distribution shifted toward the anterior-left quadrant, which was observed in 9 of 14 patients (64.3%). Overall, 9 of 14 patients (64.3%) changed quadrants after sling use. When quadrant classifications were simplified according to ML orientation, rightward trunk sway was observed in 9 of 14 patients (64.3%) during walking WO sling, decreasing to 4 of 14 patients (28.6%) during W Sling walking. Conversely, leftward trunk sway increased from 5 of 14 patients (35.7%) to 10 of 14 patients (71.4%). Among the 9 patients who initially demonstrated rightward sway, 6 (66.7%) shifted toward leftward sway following sling use. In the AP direction, anterior sway remained the predominant pattern under both walking conditions, increasing from 10 of 14 patients (71.4%) WO sling to 13 of 14 patients (92.9%) W Sling walking. Of the 4 patients who initially exhibited posterior sway, 3 (75.0%) shifted to an anterior sway orientation following sling use. Inspection according to the affected side showed that the observed leftward redistribution was not consistently associated with the paretic side. During W Sling walking, leftward sway was observed in 5 of 6 patients (83.3%) with right-sided hemiparesis and in 5 of 7 patients (71.4%) with left-sided hemiparesis, suggesting that the directional redistribution occurred regardless of the affected side.

In the ML direction, rightward sway magnitudes were largely comparable between conditions and remained close to the healthy reference threshold. In contrast, leftward sway demonstrated greater variability during W Sling walking, with a broader distribution and higher upper-range values compared with WO Sling walking. These findings suggest that arm sling use may influence ML compensation strategies, particularly by increasing leftward trunk deviation in certain individuals. The participant-level trajectories further demonstrated that the response to sling use was heterogeneous. Several participants exhibited marked shifts in sway orientation between conditions, whereas others showed relatively stable sway patterns. Collectively, these findings suggest that arm sling support modifies ML postural compensation during walking, resulting in a redistribution of trunk sway direction without a consistent reduction in overall sway magnitude.

## 4. Discussion

This study described the development and preliminary clinical application of SwayTracker, a wearable IMU-based system designed for dynamic trunk sway assessment. The proposed framework combines sternum-mounted inertial sensing, real-time signal processing, and polar-coordinate-based sway modeling to provide quantitative characterization of postural control during static and dynamic ADL. In addition to demonstrating excellent agreement with an established commercial balance assessment system, the findings showed that the proposed methodology could identify clinically meaningful differences in trunk sway behavior between healthy individuals and stroke patients under different walking conditions.

A primary finding of the study was the excellent agreement observed between SwayTracker-derived sway measurements and the Tecnobody ProKin Trunk Sensor (Tecnobody, Bergamo, Italy). Very strong correlations obtained for both AP and ML sway parameters support the concurrent validity of the proposed wearable system and indicate that trunk orientation measurements obtained from the sternum can provide an accurate representation of dynamic postural behavior during walking. These findings are consistent with previous studies reporting acceptable validity and reliability of wearable IMU systems for balance assessment, postural sway quantification, and trunk motion analysis [24,25,26]. Unlike laboratory-based motion capture systems and force platforms, which typically require dedicated facilities, specialized personnel, and substantial infrastructure, the proposed platform enables objective postural control assessment using a lightweight and portable architecture that can be deployed in routine clinical environments and potentially extended to home- and community-based monitoring applications [27,28,29].

The healthy control analysis provided normative reference values for trunk sway during self-selected walking and demonstrated that healthy individuals may exhibit substantial variability in sway direction despite maintaining relatively low overall sway magnitudes. Although the participants were distributed across multiple quadrants of the polar coordinate map, most remained clustered near the origin. This observation suggests that directional variability alone should not necessarily be interpreted as impaired balance, as postural control involves adaptive multidirectional sway strategies rather than strict directional consistency [28,30]. Rather, the overall magnitude of trunk deviation may represent a more clinically meaningful indicator of altered trunk sway behavior during walking, as larger sway amplitudes have consistently been associated with reduced postural control and postural control performance [24,26]. The normative reference thresholds established in the present study therefore provide an objective framework for distinguishing physiological variability from potentially pathological trunk sway behavior.

Relative to the normative reference distribution established from the healthy participants, the stroke survivors generally exhibited larger trunk sway magnitudes. It should be emphasized that the healthy participants were included to establish methodological reference values and a normative sway distribution rather than to serve as an age-matched clinical control group. Therefore, observations involving the healthy cohort should be interpreted as methodological contextualization rather than direct disease-related comparisons. The predominance of anterior trunk sway observed in both walking conditions may reflect compensatory postural strategies commonly reported after stroke. Previous studies have shown that stroke-related neurological impairments frequently lead to asymmetrical weight transfer, impaired trunk control, altered postural regulation, and reduced balance capacity during gait, resulting in compensatory movement strategies aimed at maintaining postural control [26,31]. These deficits frequently lead to excessive trunk displacement and increased reliance on compensatory movements to maintain forward progression. The elevated sway magnitudes observed in the present cohort are therefore consistent with the impaired dynamic balance control typically reported in hemiparetic gait.

One of the most interesting findings of the study was that arm sling use appeared to modify the directional organization of trunk sway without substantially changing overall sway magnitude. During WO Sling walking, trunk sway was predominantly concentrated within the anterior-right quadrant, whereas W Sling walking produced a noticeable shift toward the anterior-left quadrant. Despite this directional redistribution, composite sway radius values remained largely unchanged between conditions. These findings suggest that arm sling support may influence the strategy used to control posture rather than directly reducing instability. The observed shift in mediolateral sway orientation may be related to altered upper-extremity loading, changes in trunk muscle recruitment, or modifications in weight-transfer behavior induced by stabilization of the affected upper limb. The clinical meaning of the observed mediolateral directional shift requires cautious interpretation. Post-stroke gait is frequently characterized by asymmetric trunk motion, impaired weight transfer, and reduced movement toward the paretic side, with lateral trunk displacement often serving as a compensatory strategy to maintain progression and postural control [11,12]. Such compensations may be functionally useful when they facilitate limb advancement or reduce the perceived threat of instability; however, excessive or persistent lateral trunk deviation may also indicate impaired trunk control, mechanical inefficiency, or reliance on the non-paretic side. In the present study, arm sling use modified the mediolateral organization of trunk sway but did not reduce the composite sway magnitude. Therefore, the directional shift should not be interpreted as evidence of improved balance by itself. Rather, it may represent an individualized reorganization of postural control associated with altered upper-limb support and trunk–pelvis coordination. Whether this adaptation is beneficial or detrimental likely depends on the direction of sway relative to the paretic side, the degree of weight-bearing symmetry, walking performance, and the patient’s baseline motor impairment. Future studies should therefore relate directional sway changes to paretic-side orientation, plantar-pressure distribution, step symmetry, energy cost, and fall-related outcomes.

Previous investigations examining arm sling use after stroke have primarily focused on spatiotemporal gait characteristics, including walking speed, cadence, stride length, and gait symmetry, as well as clinical and functional balance assessments such as the Berg Balance Scale and Functional Reach Test [12,13,31]. Although some studies have reported improvements in gait performance or subjective postural control with arm sling use after stroke, the biomechanical mechanisms responsible for these changes remain largely unexplored. Previous investigations have focused on clinical balance scales and spatiotemporal gait parameters, with limited attention given to dynamic trunk behavior and postural control during walking [13,30]. The present findings extend existing knowledge by demonstrating that wearable trunk sway analysis can reveal directional adaptations that may not be captured by conventional clinical outcome measures. In particular, the polar-coordinate framework provided a visual and quantitative representation of how trunk sway organization changed under sling-assisted conditions, highlighting the potential value of direction-sensitive balance metrics in rehabilitation research.

Another important observation was the substantial inter-individual variability in trunk sway responses. While several participants demonstrated marked directional shifts following sling application, others showed relatively stable sway patterns. This heterogeneity suggests that the biomechanical effects of arm sling use may depend on patient-specific factors such as severity of hemiparesis, trunk control capacity, affected side, compensatory gait strategy, and stage of recovery. Consequently, arm sling prescription may not produce uniform postural responses across all stroke patients, supporting the need for individualized assessment approaches. Wearable monitoring technologies such as SwayTracker may therefore provide clinically useful information for identifying patients who are more likely to benefit from specific upper-extremity support strategies. Moreover, although the overall magnitude of trunk sway did not change with arm sling use, a subtle increase in anterior trunk inclination was observed in several participants. One possible explanation is the mechanical configuration of the shoulder sling itself. Because the sling is suspended by a strap passing over the neck and shoulder, it may introduce a slight anterior pulling force on the upper trunk, encouraging a more forward-oriented posture during walking. This interpretation is also consistent with subjective feedback obtained from several participants, who reported feeling that the sling pulled them slightly forward while ambulating. Although these observations were not formally quantified, they suggest that the design and suspension characteristics of upper extremity slings may influence trunk orientation independently of their intended role in supporting the affected limb. Future studies incorporating three-dimensional trunk kinematics and comparisons between different sling designs may help clarify these biomechanical effects. These findings suggest that arm sling use may modify patient-specific postural compensation strategies rather than producing a uniform biomechanical response. The considerable inter-individual variability observed in directional trunk sway may reflect differences in trunk control, hemiparetic involvement, upper-extremity stabilization requirements, and weight-transfer strategies.

From a translational perspective, the proposed polar-coordinate-based sway metrics are intended to complement, rather than replace, existing clinical assessments of postural control. Conventional clinical scales, such as the Trunk Impairment Scale, Berg Balance Scale, Functional Gait Assessment, and Timed Up and Go, provide valuable information regarding functional performance but are largely based on ordinal scoring and observer interpretation. In contrast, the proposed wearable system provides objective quantitative measures of trunk sway magnitude and directional organization during walking. Combining these approaches may enable a more comprehensive assessment of dynamic postural control by integrating functional performance with biomechanical characterization. For example, patients presenting with similar Trunk Impairment Scale or Berg Balance Scale scores may exhibit different directional trunk sway patterns, potentially reflecting distinct compensatory strategies that are not readily detected during routine clinical examination. Furthermore, repeated SwayTracker assessments could facilitate longitudinal monitoring of postural adaptations throughout rehabilitation and may assist clinicians in evaluating individual responses to interventions such as arm sling use, balance training, or task-specific gait rehabilitation. Future studies should investigate the prognostic value of these metrics and determine whether their integration with established clinical scales improves fall-risk prediction and individualized rehabilitation planning.

It is important to note that the technical validation was performed under controlled static conditions using predefined inclination angles to isolate sensor accuracy from gait-related biological variability. Consequently, the reported agreement statistics should be interpreted as evidence of technical measurement performance rather than as a comprehensive evaluation of dynamic measurement validity during walking. Although SwayTracker was subsequently applied during overground gait assessments and generated clinically interpretable trunk sway measurements, static validation alone cannot fully account for the effects of trunk accelerations, soft tissue motion, and other movement-related factors encountered during locomotion. Therefore, future studies should establish the dynamic concurrent validity of SwayTracker by comparing its measurements with laboratory-based motion capture systems or other validated wearable reference devices during functional gait tasks.

This study has some limitations. First, although the study met the predefined sample size estimated by the a priori power analysis, the relatively small cohort may still have limited the ability to detect subtle between-condition differences, thereby increasing the possibility of Type II error. Consequently, the absence of statistically significant differences should be interpreted with appropriate caution until confirmed in larger cohorts. Moreover, the subgroup analyses according to stroke phase (acute, subacute, and chronic) and paretic side were not performed because of the limited sample size. The biomechanical effects of arm sling use may vary according to stroke chronicity, lesion characteristics, paretic side, and the severity of motor impairment. Consequently, the present findings represent the overall response of a heterogeneous stroke cohort and should not be interpreted as being equally applicable to all stroke subpopulations. Future studies with larger and clinically stratified cohorts are needed to determine whether directional trunk sway adaptations differ according to stroke phase, paretic side, or functional status. In addition, the healthy and stroke cohorts differed in age, BMI, and self-selected walking speed. These variables are known to influence trunk sway and postural control and therefore may have contributed to the observed between-group differences. Because the primary objective of this study was to establish a methodological reference framework and to evaluate within-subject changes associated with arm sling use, the principal analyses focused on comparisons within the stroke cohort rather than direct inference between the healthy and stroke participants. Accordingly, the healthy participant data should be interpreted as a reference dataset rather than an age-matched control population. Future studies involving larger age-matched cohorts will allow multivariable analyses, including ANCOVA or mixed-effects modeling, to further isolate the independent effects of stroke on trunk sway. Furthermore, the reference dataset was derived exclusively from healthy young adults and therefore should not be considered representative of middle-aged or older populations. Consequently, the proposed reference thresholds are intended as methodological reference values for the present analytical framework rather than age-specific normative standards for clinical interpretation. Future studies should establish age-stratified normative datasets by recruiting healthy participants across different decades of life, thereby improving the external validity and clinical applicability of the proposed trunk sway assessment framework. Second, healthy participants were substantially younger than stroke patients, which limits direct age-matched comparisons of trunk sway behavior. Third, the present study evaluated only the immediate effects of arm sling use during a single walking session and therefore reflects acute biomechanical responses rather than long-term adaptations. Consequently, it remains unclear whether the observed directional reorganization of trunk sway would persist following prolonged sling use or structured rehabilitation, or whether these immediate changes would translate into sustained improvements in gait performance, trunk control, balance, or functional recovery. Therefore, the present findings should not be interpreted as evidence of long-term therapeutic benefit. Future longitudinal studies incorporating repeated assessments over extended intervention periods are warranted to determine the persistence and clinical significance of these biomechanical adaptations. In addition, the study focused primarily on trunk orientation measures and did not simultaneously evaluate lower-extremity kinematics, muscle activity, or kinetic variables that may contribute to the observed postural adaptations. Another limitation is that all the gait assessments were performed under standardized treadmill walking conditions. Although treadmill-based protocols provide a controlled environment that minimizes variability and improves measurement consistency, gait biomechanics and trunk sway characteristics may differ from those observed during overground walking. Consequently, the present findings should not be directly generalized to unrestricted ward or community ambulation. Future studies should evaluate the proposed SwayTracker system during overground walking and in real-world environments to establish its ecological validity and determine the applicability of the proposed sway metrics under everyday walking conditions. Another important limitation is that the healthy and stroke groups were not age-matched and walked at substantially different self-selected speeds. Because both age and walking speed influence postural control and trunk sway behavior, these factors may have contributed to the observed between-group differences. Therefore, the healthy participant data should be interpreted as a methodological reference dataset for the proposed sway metrics rather than normative values directly applicable to the stroke population. The primary purpose of the healthy cohort was to establish reference characteristics for the newly proposed analytical framework, whereas the principal clinical analyses focused on within-subject comparisons between walking with and without arm sling support. Future studies should include age-matched healthy controls walking at comparable speeds to further validate the proposed reference framework. Additionally, to address the potential confounding effect of walking speed, the healthy participants additionally completed standardized walking trials at 1 km/h, and these data were used to derive complementary reference thresholds for trunk sway interpretation. Nevertheless, future studies should include age-matched healthy controls walking at comparable speeds to further validate the proposed reference framework. Finally, the present study focused on concurrent validity and did not evaluate test–retest reliability or repeatability of the proposed wearable system. Therefore, although the current findings support the preliminary concurrent validity of SwayTracker, they do not establish its measurement of postural control across repeated sessions. Future studies should investigate intra- and inter-session reliability using test–retest protocols, intraclass correlation coefficients (ICCs), and additional measurement error indices to determine the suitability of the system for longitudinal rehabilitation monitoring and treatment-response evaluation.

Despite these limitations, the study demonstrates the feasibility of combining wearable IMU technology with polar-coordinate-based sway analysis for dynamic balance assessment during walking. The proposed methodology provides clinically interpretable information regarding both the magnitude and direction of trunk sway and may complement existing gait and balance evaluation approaches. Future studies involving larger cohorts, longitudinal follow-up, and additional neurological populations are warranted to further establish the clinical utility of wearable trunk sway monitoring and to determine whether direction-specific sway metrics can serve as sensitive indicators of rehabilitation outcomes and fall risk.

## Figures and Tables

**Table 1:** Participant characteristics of healthy controls and stroke patients.

Characteristic	Healthy Controls (*n* = 15)	Stroke Patients (*n* = 14)	*p*-Value
Age (years)	20.9 ± 1.1	57.6 ± 9.5	<0.001
Sex (M/F)	6/9	10/4	0.139
Height (cm)	170.7 ± 9.3	173.5 ± 8.2	0.576
Weight (kg)	62.5 ± 13.4	77.4 ± 14.1	0.089
BMI (kg/m^2^)	21.3 ± 2.9	25.7 ± 4.4	0.049
Time Since Stroke (months)	—	14.7 ± 20.0	—
Affected Side (Right/Left/Bilateral)	—	6/7/1	—
Walking Speed (km/h)	2.8 ± 0.8	0.7 ± 0.4	<0.001

Group comparisons were performed using Mann–Whitney U tests for continuous variables and Fisher’s exact test for sex distribution. BMI: body mass index. Statistical significance was set at *p* < 0.05.

**Table 2:** Polar Coordinate Representations of Stroke Patients During Walking Without Sling Support.

Participant	Stroke Category	Affected Side	Roll Direction	Roll Magnitude (°)	Pitch Direction	Pitch Magnitude (°)	Theta (°)	Radius (°)
SP1	Hemorrhagic stroke	Right	Right	8.90	Forward	11.50	52.26	14.54
SP2	Hemorrhagic stroke	Left	Left	6.00	Backward	6.25	226.17	8.66
SP3	Hemorrhagic stroke	Right	Left	4.30	Backward	10.31	247.36	11.17
SP4	Ischemic stroke	Bilateral	Right	11.00	Forward	15.06	53.86	18.65
SP5	Ischemic stroke	Left	Right	7.50	Backward	7.87	313.62	10.87
SP6	Hemorrhagic stroke	Left	Right	12.20	Forward	12.38	45.42	17.38
SP7	Hemorrhagic stroke	Right	Right	5.30	Forward	21.06	75.87	21.72
SP8	Stroke, unspecified	Right	Right	4.00	Forward	8.44	64.64	9.34
SP9	Ischemic stroke	Right	Left	8.80	Forward	10.75	129.30	13.89
SP10	Ischemic stroke	Left	Left	9.20	Forward	7.81	139.67	12.07
SP11	Ischemic stroke	Left	Left	8.60	Forward	26.62	107.90	27.97
SP12	Hemorrhagic stroke	Left	Right	8.30	Forward	18.37	65.69	20.16
SP13	Hemorrhagic stroke	Left	Right	10.90	Forward	17.00	57.33	20.19
SP14	Stroke, unspecified	Right	Right	3.80	Backward	7.50	296.87	8.41
μ ± SD (°)	-	-	-	7.77 ± 2.72	-	12.92 ± 5.98	Circular μ ± Circular SD (°): 69.49 ± 79.15	15.36 ± 5.83

SD, standard deviation. Roll and Pitch directions indicate the dominant mediolateral and anteroposterior trunk sway directions, respectively. Theta (θ) represents the polar direction angle (°), and Radius represents the resultant trunk sway magnitude (°). Theta values are presented as circular mean ± circular standard deviation.

**Table 3:** Paired comparison of W Sling and WO Sling conditions in stroke participants.

Outcome	Radius (°)	ML/Roll Magnitude (°)	AP/Pitch Magnitude (°)	Theta (°, Circular)	Joint Signed ML/AP Vector
WO Sling	15.36 ± 5.83	7.77 ± 2.72	12.92 ± 5.98	74.50 ± 80.33	-
W Sling	15.35 ± 4.59	8.53 ± 3.55	12.34 ± 4.49	110.20 ± 41.97	-
W-WO Change	−0.01	0.76	−0.59		5.79
95% CI	−2.31 to 2.30	−0.93 to 2.45	−3.11 to 1.93	-	-
Test Statistics	t(13) = −0.006	t(13) = 0.968	t(13) = −0.503	T^2^(2, 12) = 6.633	T^2^(2, 12) = 2.885
*p*	0.996	0.351	0.623	0.084	0.300
Effect/Vector Summary	dz = −0.001	dz = 0.259	dz = −0.135	Mean unit-vector distance = 0.510	ML −5.0429; AP 2.8400

Linear outcomes are arithmetic mean ± SD and were compared with paired t-tests; changes are W Sling minus WO Sling. Theta is reported as circular mean ± circular SD and was compared using a paired Hotelling test on sine/cosine unit-vector components. The joint signed ML/AP row tests the paired two-dimensional component change. All the analyses include SP1–SP14 (*n* = 14).

**Table 4:** Polar Coordinate Representation of Stroke Patients During Walking with Sling Support.

Participant	Stroke Category	Affected Side	Roll Direction	Roll Magnitude (°)	Pitch Direction	Pitch Magnitude (°)	Theta (°)	Radius (°)
SP1	Hemorrhagic stroke	Right	Left	11.50	Forward	7.19	147.99	13.56
SP2	Hemorrhagic stroke	Left	Left	5.50	Forward	8.38	123.28	10.02
SP3	Hemorrhagic stroke	Right	Left	5.00	Forward	16.31	107.04	17.06
SP4	Ischemic stroke	Bilateral	Right	6.40	Forward	11.63	61.18	13.27
SP5	Ischemic stroke	Left	Left	10.50	Backward	7.94	217.10	13.16
SP6	Hemorrhagic stroke	Left	Left	14.50	Forward	10.56	143.94	17.94
SP7	Hemorrhagic stroke	Right	Right	11.70	Forward	16.69	54.97	20.38
SP8	Stroke, unspecified	Right	Left	2.80	Forward	7.94	109.42	8.42
SP9	Ischemic stroke	Right	Left	6.10	Forward	9.88	121.69	11.61
SP10	Ischemic stroke	Left	Left	11.30	Forward	11.25	135.13	15.95
SP11	Ischemic stroke	Left	Right	10.50	Forward	15.25	55.45	18.52
SP12	Hemorrhagic stroke	Left	Left	11.50	Forward	21.50	118.14	24.38
SP13	Hemorrhagic stroke	Left	Right	8.00	Forward	18.37	66.47	20.04
SP14	Stroke, unspecified	Right	Left	4.10	Forward	9.81	112.68	10.63
μ± SD(°)	-	-	-	8.53 ± 3.55	-	12.34 ± 4.49	Circular μ ± Circular SD (°): 110.20 ± 41.97	15.35 ± 4.59

SD, standard deviation. Roll and Pitch directions indicate the dominant mediolateral and anteroposterior trunk sway directions, respectively. Theta (θ) represents the polar direction angle (°), and Radius represents the resultant trunk sway magnitude (°). Theta values are presented as circular mean ± circular standard deviation.

**Table 5:** Paired Comparison of Trunk Sway Characteristics During Walking WO and W Sling.

Variable	WO Sling (Mean ± SD)	W Sling (Mean ± SD)	t	*p*
ML sway (Roll magnitude °)	7.77 ± 2.72	8.53 ± 3.55	0.968	0.351
AP sway (Pitch magnitude °)	12.92 ± 5.98	12.34 ± 4.49	−0.503	0.623
Theta (°)	69.49 ± 79.15	110.20 ± 41.97	−0.947	0.361
Combined sway magnitude (Radius °)	15.36 ± 5.83	15.35 ± 4.59	−0.005	0.996

WO, without; W, with; ML, mediolateral; AP, anteroposterior; SD, standard deviation. Theta represents the polar direction angle of trunk sway (°). Values are expressed as circular means and standard deviations.

**Table 6:** Patient-Level Quadrant-Based Trunk Sway Direction Transitions According to the Affected Side WO Sling and W Sling Walking Conditions.

Participant	Stroke Category	Affected Side	WO Sling Quadrant	W Sling Quadrant
SP1	Hemorrhagic stroke	Right	Anterior Right	Anterior Left
SP2	Hemorrhagic stroke	Left	Posterior Left	Anterior Left
SP3	Hemorrhagic stroke	Right	Posterior Left	Anterior Left
SP4	Ischemic stroke	Bilateral	Anterior Right	Anterior Right
SP5	Ischemic stroke	Left	Posterior Right	Posterior Left
SP6	Hemorrhagic stroke	Left	Anterior Right	Anterior Left
SP7	Hemorrhagic stroke	Right	Anterior Right	Anterior Right
SP8	Stroke, unspecified	Right	Anterior Right	Anterior Left
SP9	Ischemic stroke	Right	Anterior Left	Anterior Left
SP10	Ischemic stroke	Left	Anterior Left	Anterior Left
SP11	Ischemic stroke	Left	Anterior Left	Anterior Right
SP12	Hemorrhagic stroke	Left	Anterior Right	Anterior Left
SP13	Hemorrhagic stroke	Left	Anterior Right	Anterior Right
SP14	Stroke, unspecified	Right	Posterior Right	Anterior Left

## Data Availability

Data set is available upon request.

## Abbreviations

The following abbreviations are used in this manuscript:

ADLs	Activities of Daily Living
AP	Anteroposterior
BBS	Berg Balance Scale
BMI	Body Mass Index
CV	Coefficient of Variation
ICC	Intraclass Correlation Coefficient
IMU	Inertial Measurement Unit
I^2^C	Inter-Integrated Circuit
ML	Mediolateral
P95	95th Percentile
PDF	Portable Document Format
SD	Standard Deviation
SEM	Standard Error of Measurement
P	Healthy Participant
SP	Stroke Participant
TUG	Timed Up and Go
W Sling	Walking with Sling
WO Sling	Walking without Sling
